# Exendin-4 Protects against Hyperglycemia-Induced Cardiomyocyte Pyroptosis via the AMPK-TXNIP Pathway

**DOI:** 10.1155/2019/8905917

**Published:** 2019-12-09

**Authors:** Hong Wei, Rui Bu, Qinghui Yang, Jing Jia, Tao Li, Qiuping Wang, Yanjun Chen

**Affiliations:** ^1^Department of Cardiology, The Fourth Affiliated Hospital of Harbin Medical University, Harbin, China; ^2^Department of Cardiology, Peking University Shenzhen Hospital, Shenzhen, China

## Abstract

Diabetic cardiomyopathy is a common cardiac condition in patients with diabetes mellitus, which results in cardiac hypertrophy and subsequent heart failure. Chronic inflammation in the diabetic heart results in loss of cardiomyocytes and subsequentially cardiac dysfunction. Accumulated evidence implicated pyroptosis as a vital contributor to the hyperglycemia-induced cardiac inflammatory response. Exendin-4, a GLP analog, promotes survival of cardiomyocytes in cardiovascular diseases, including diabetic cardiomyopathy. However, the role of Exendin-4 in cardiac pyroptosis remains to be elucidated. Our study revealed that Exendin-4 treatment protected against heart remolding and dysfunction and attenuated cardiac inflammation in high-fat diet-fed rats. The activity of caspase-1 and production of pyroptotic cytokines were significantly inhibited by Exendin-4 treatment in the diabetic heart and in high glucose-treated cardiomyocytes as well. In an effort to understand the signaling mechanisms underlying the antipyroptotic property of Exendin-4, we found that blockade of AMPK, an oxidative stress sensor, activity diminished the antipyroptotic property of Exendin-4. Phosphorylation of AMPK resulted in degeneration of TXNIP that promoted the activation of the NLRP3 inflammasome. Exendin-4 treatment decreased the protein level of TXNIP. Moreover, RNA silencing of TXNIP mimicked the antipyroptotic actions of Exendin-4. These findings promoted us to propose a new signaling pathway mediating cardioprotective effect of Exendin-4 under hyperglycemic conditions: Exendin-4 → ROS↓ → pAMPK↑ → TXNIP↓ → caspase-1↓ → IL-1*β* and IL-18↓ → pyroptosis↓. In general, our study identified Exendin-4 as a pyroptotic inhibitor protecting against hyperglycemia-induced cardiomyocyte pyroptosis via the AMPK-TXNIP pathway.

## 1. Introduction

Diabetes mellitus (DM) is a group of metabolic disorders characterized by hyperglycemia. Patients with diabetes suffer from cardiovascular diseases 2 to 4 times likely than individuals without diabetes [1]. Diabetic cardiomyopathy (DCM), the leading diabetic complication, is a critical cause of fatalities in chronic DM patients. DCM is defined by abnormal myocardial structure and cardiac function in the absence of coronary atherosclerosis and hypertension [2]. In DCM, chronic cardiac inflammation is characterized contributing to loss of cardiomyocytes that results in impaired systolic function [3, 4]. However, the mechanism and medical treatment remain to be elucidated.

Accumulating evidence implicated pyroptosis as a critical contributor to myocardial inflammation in DCM [5–9]. Pyroptosis, the proinflammatory programmed cell death, is mechanistically different from necrosis and apoptosis [10]. Pyroptosis results in the release of cytokines that activate proinflammatory immune cells [11, 12]. In the diabetic heart, hyperglycemia induces high level of reactive oxygen species (ROS), which stimulates activation of the nucleotide-binding oligomerization domain-like receptor pyrin domain containing (NLRP) 3 inflammasome [7]. Caspase-1, the specific pyroptotic caspase, is recruited to the inflammasome and activated via autoprocessing [13]. Activated caspase-1 subsequentially processes interleukin- (IL-) 1*β*, IL-18, and pore-forming protein, gasdermin D (GSDMD), to activated forms, ultimately resulting in robust cell lysis with rapid release of proinflammatory cytokines [11, 12]. As the cores of the signal cascade, NLRP3 and caspase-1 are identified as pyroptotic markers and pharmaceutic targets of DCM [14].

Glucagon-like peptide-1 (GLP-1) is identified with metabolic effects for glycemic control in type 2 DM [15]. Furthermore, GLP-1 is involved in cardiovascular physiology and protects cardiomyocytes against hyperglycemia-induced toxicity in DCM [16]. Exendin-4, a GLP analog, has a longer half-life than GLP-1 through evading clearance by dipeptidyl peptidase IV (DPP-4) [17]. In diabetic rodent models, Exendin-4 treatment improved cardiac function and glucose uptake [18]. Pretreatment of Exendin-4 also reduces the infarct area in the rat heart after ischemia reperfusion [19]. In these models, previous studies focused on the protective effect of Exendin-4 on cardiomyocyte apoptosis. However, the role of Exendin-4 in pyroptosis is largely unknown. The aim of this study is to investigate the potential benefits of Exendin-4 in hyperglycemia-induced cardiac remolding, inflammation, and cardiomyocyte pyroptosis and clarify the underlying molecular mechanisms.

## 2. Materials and Methods

### 2.1. Animals

C57BL/6J mice were obtained from the Model Animal Research Center (MARC) of Nanjing University (Nanjing, China). Animals were maintained in an SPF-grade laminar-flow housing apparatus under controlled temperature, humidity, and 12 h light/dark regimen. The experimental animal facility has been accredited by the AAALAC (Association for Assessment and Accreditation of Laboratory Animal Care International), and the IACUC (Institutional Animal Care and Use Committee) of Model Animal Research Center of Nanjing University approved all animal protocols used in this study.

### 2.2. Chemicals

Exendin-4 is purchased from MedChemExpress (MCE, HY-13443, USA) and dissolved in PBS with 10% DMSO. Compound C (CC) was purchased from Sigma-Aldrich Chemical (CAS 866405-64-3, Germany) and dissolved in PBS.

### 2.3. High-Fat Diet-Induced Type 2 Diabetic Mouse Model

For the high-fat diet-induced type 2 diabetic mouse study, 21 mice were randomly distributed to two groups fed a control diet (CON) (10% kcal fat, 70% kcal carbohydrate, and 23% kcal protein with a total caloric value of 3.85 kcal/gm, *n* = 7) or a high-fat diet (consisting of 45% kcal fat, 35% kcal carbohydrate, and 20% kcal protein with a total caloric value of 4.73 kcal/gm, *n* = 14). After a 16-week dietary intervention, the high-fat diet-fed group was then randomly subdivided to receive Exendin-4 (HFD+EXE) (25 nmol/kg/d, *n* = 7) or normal saline (HFD) by intraperitoneal injection during the light cycle. For 8-week administration of Exendin-4, the animals were sacrificed for cardiac histology and inflammation analysis.

### 2.4. Intraperitoneal Glucose Tolerance Test (IPGTT)

The glucose tolerance test (*n* = 7 each group) was measured in three groups of mice 7 days before sacrifice. Briefly, the IPGTT was conducted after an overnight fast (12–16 h). Mice were injected with 40% glucose (2 g/kg body weight). Blood glucose was measured from the tail tip using a glucose meter (OMRON, Japan) at 0, 15, 30, 45, 60, 90, and 120 min.

### 2.5. Echocardiography

Echocardiography was performed 3 days before sacrifice. The mice in each group were anesthetized with isoflurane. Transthoracic two-dimensional M-mode echocardiography and pulsed-wave Doppler spectral tracings were obtained using the Vevo 2100 Imaging System (VisualSonics, Canada). The percentages of ejection fraction (EF%) were measured using M-mode tracings. The percentage of fractional shortening (FS) was calculated according to the following formula [(LVDD − LVSD)/LVDD] × 100%.

### 2.6. Histology

For 24-week dietary intervention, hearts were isolated and fixed in 4% paraformaldehyde for 4 hours followed by gradual dehydration. Then, the heart tissues were embedded in paraffin and cut into 6 *μ*m sections. Masson's trichrome staining was performed as MARC SOP, and the samples were imaged with an Olympus inverted microscope (Olympus BX51, Japan).

### 2.7. Immunofluorescence and Immunohistochemistry

Immunofluorescence staining of WGA was performed as previously reported. Briefly, the hearts were fixed with 4% paraformaldehyde for 2 hours on ice and embedded in optimum cutting temperature compound (OCT, Leica, 4853, Germany). The frozen samples were cut into 10 *μ*m sections. The sections were rinsed with PBS and incubated with Wheat Germ Agglutinin (WGA; Thermo Fisher, W11261, USA) and 4′,6-diamidino-2-phenylindole (DAPI; Jackson ImmunoResearch, USA) at 4°C for 30 min. FLICA staining was performed according to the manufacturer's instructions (ICT097, Bio-Rad, USA). The fluorescent images were captured with a ZEISS LM780 laser scanning confocal microscope (Zeiss, Germany).

Immunohistochemistry was performed to examine the level of pyroptotic proteins in the diabetic myocardium. The preparation of section samples was the same as in histological analysis. After antigen retrieval with heating, specimens were incubated with primary antibodies against cleaved caspase-1 (Cell Signaling Technology, #4199, MA, USA), NLRP3 (Novus Biologicals, NBP2-12446, USA), IL-1*β* (Abcam, ab2105, UK), and IL-18 (Abcam, ab71495, UK) at 4°C overnight. After incubation with secondary antibodies, the sections were stained with diaminobenzidine and imaged with an Olympus inverted microscope (Olympus BX51, Japan).

### 2.8. Isolation and Culture of Primary Cardiomyocytes

Primary cardiomyocytes were isolated from 1- to 3-day-old neonatal C57BL/6 mice via collagenase digestion according to the manufacturer's protocol (Worthington, USA). Briefly, cardiac tissues were rinsed with HBSS and sequentially digested by pancreatin and collagenase type II without Ca^2+^. The isolated cells were resuspended in Ca^2+^ containing L15 medium with oxygen awaking. Cardiomyocytes were filtered and purified by differential plating, and 0.1 mM 5-bromo-2-deoxyuridine (BrdU; Sigma-Aldrich, B5002, Germany) was added to the medium to prevent the proliferation of nonmyocytes. Dulbecco's modified Eagle's medium (DMEM) with 10% fetal bovine serum (FBS) and 1% penicillin/streptomycin (Hyclone, USA) were used to culture the cardiomyocytes, which were divided into four groups and treated with low glucose (5 mM glucose, CON) or high glucose (40 mM glucose, HG) with or without Exendin-4 (25 nM, HG+EXE) and CC (10 *μ*M, HG+EXE+CC) for 24 h. All cells were incubated at 37°C in humidified air with 5% CO_2_.

### 2.9. Gene Transfection

Cells were transfected using X-tremeGENE siRNA Transfection Reagent (Roche, 04476093001, Germany) according to the manufacturer's instructions. The siRNA-targeting mouse TXNIP and nontargeting siRNA were each synthesized by GenePharma (Shanghai, China). The TXNIP siRNA sense sequence is 5′-UGGUCACGUCGAAAUGAAUTT-3′, and the antisense sequence is 5′-TTGACACGUGCUCCCUACGUG-3′. Measurements were performed 24 h after the transfection.

### 2.10. Reverse Transcriptase qPCR

The animals were euthanized, and the hearts were dissected and frozen in liquid nitrogen. Total RNAs from cultured neonatal cardiomyocytes or heart tissues were extracted using 1 ml of TRIzol reagent (Invitrogen, USA) according to the manufacturer's instructions. cDNA synthesis was performed using the SuperScript III Reverse Transcriptase Kit (Thermo Fisher, 18080044, USA) according to the manufacturer's instructions. The SYBR Green PCR Master Mix Kit (Applied Biosystems, 4309155, USA) was used to quantify the relative mRNA levels of IL-1*β*, IL-6, IL-10, TNF-*α*, ICAM-1, VCAM-1, caspase-1, NLRP3, and TXNIP. Real-time PCR was performed with the 7500 FAST Real-Time PCR System (Applied Biosystems, USA) for 40 cycles, with GAPDH serving as internal controls. The following primers were used in the study:

- IL-1*β*: forward, 5′-CCCTGCAGCTGGAGAGTGTGG-3′, and reverse, 5′-TGTGCTCTGCTTGAGAGGTGCT-3′
- IL-6: forward, 5′-TACCACTTCACAAGTCGGAGGC-3′, and reverse, 5′-CTGCAAGTGCATCATCGTTGTTC-3′
- IL-10: forward, 5′-CGGGAAGACAATAACTGCACCC-3′, and reverse, 5′-CGGTTAGCAGTATGTTGTCCAGC-3′
- TNF-*α*: forward, 5′-GGTGCCTATGTCTCAGCCTCTT-3′, and reverse, 5′-GCCATAGAACTGATGAGAGGGAG-3′
- TGF-*β*: forward, 5′-CGAAGCGGACTACTATGCTAAAGAG-3′, and reverse, 5′-TGGTTTTCTCATAGATGGCGTTG-3′
- ICAM-1: forward, 5′-GTGGCGGGAAAGTTCCTG-3′, and reverse, 5′-CGTCTTGCAGGTCATCTTAGGAG-3′
- VCAM-1: forward, 5′-AGTTGGGGATTCGGTTGTTC-3′, and reverse, 5′-CATTCCTTACCACCCCATTG-3′
- Caspase-1: forward, 5′-ACACGTCTTGCCCTCATTATCT-3′, and reverse, 5′-ATAACCTTGGGCTTGTCTTTCA-3′
- NLRP3: forward, 5′-TCACAACTCGCCCAAGGAGGAA-3′, and reverse, 5′-AAGAGACCACGGCAGAAGCTAG-3′
- TXNIP: forward, 5′-GGCCGGACGGGTAATAGTG-3′, and reverse, 5′-AGCGCAAGTAGTCCAAAGTCT-3′
- GAPDH: forward, 5′-CATCACTGCCACCCAGAAGACTG-3′, and reverse, 5′-ATGCCAGTGAGCTTCCCGTTCAG-3′

### 2.11. ELISA

Protein extraction was purified from heart tissue (CON, HFD and HFD+EXE) and culture medium of primary cardiomyocytes (CON, HG and HG+EXE). The contraction of pro-inflammatory cytokine (IL-1*β* (MLB00C), IL-6 (M6000B), IL-18 (DY122-05), TNF-*α* (SMTA00B) and TGF-*β* (SMB100B)) was determined by ELISA kit (R&D Systems, USA) according to the manufacturer's instructions. For IL-10 (M1000B), the protein was collected from 6-week Exendin-4-treated hearts. The insulin ELISA was performed according to the manufacturer's instructions (Thermo Fisher, EMINS, USA).

### 2.12. Caspase-1 Activity Assay

Caspase-1 activity was determined using the Caspase-1 Assay Kit (fluorometric) from Abcam (ab39412, UK) according to the manufacturer's instructions. Briefly, cells were lysed and the nuclei and organelles were removed by centrifugation at 20,000*g*. 50 *μ*g of total cytosolic protein was used to assess cytosolic caspase activity. Cell homogenates were incubated up to 4 hours at 37°C with corresponding caspase substrate conjugated to the chromophore p-nitroanilide. Cleavage of the substrate was quantified spectrophotometrically at 405 nm using a plate reader (Bio-Rad, USA).

### 2.13. Western Blotting

The Western blot assay was conducted as previously described. Briefly, cell lysates prepared in standard cell lysis buffer were separated in 10% SDS-polyacrylamide, and proteins were transferred to PVDF membranes (Millipore, USA). The membranes were subsequently blocked by 5% BSA dissolved in PBS for 1 h and probed with primary antibodies against caspase-1 (Novus Biologicals, NBP1-45433, USA), NLRP3 (Novus Biologicals, NBP2-12446, USA), ASC (Cell Signaling Technology, #67824, USA), TXNIP (Novus Biologicals, NBP1-54578), AMPK (Cell Signaling Technology, #2532, USA), and p-AMPK (Cell Signaling Technology, #2535, USA) and secondary HRP-conjugated antibodies. GAPDH (Santa Cruz Biotechnology, sc-32233, USA) was used as an internal control. Western blotting bands were quantified using the Odyssey Infrared Imaging System (Tanon, China) by measuring band intensity (area × OD) and developed using the ECL detection reagent (Vazyme, China).

### 2.14. ROS Determination

Intracellular ROS level was labeled by dichloro-dihydro-fluorescein diacetate (DCFH-DA) assays (Sigma-Aldrich, D6883 Germany). Briefly, cells were incubated with 50 *μ*M DCFH-DA at 37°C for 30 min in darkness. Then, the cells were washed twice using cold PBS and harvested for fluorescence-activated cell sorting.

### 2.15. Statistical Analysis

Statistical details of individual experiments, including the number of samples, mean values, standard error of the mean (SEM), and *p* values derived from two-tailed *t*-tests, are described in the figure legends and specified in the figures. Statistical analyses were performed using the GraphPad Prism software. *p* values ≤ 0.05 was considered statistically significant. For comparison between two treatments, Student's two-tailed t-test was used. Asterisk coding in figures is as follows: ^∗^*p* ≤ 0.05, ^∗∗^*p* ≤ 0.01, ^∗∗∗^*p* ≤ 0.005, and ^∗∗∗∗^*p* ≤ 0.001.

## 3. Results

### 3.1. Exendin-4 Ameliorates Glucose Metabolic Disorder in High-Fat Diet-Induced Rodent Models

**Figure 1:**
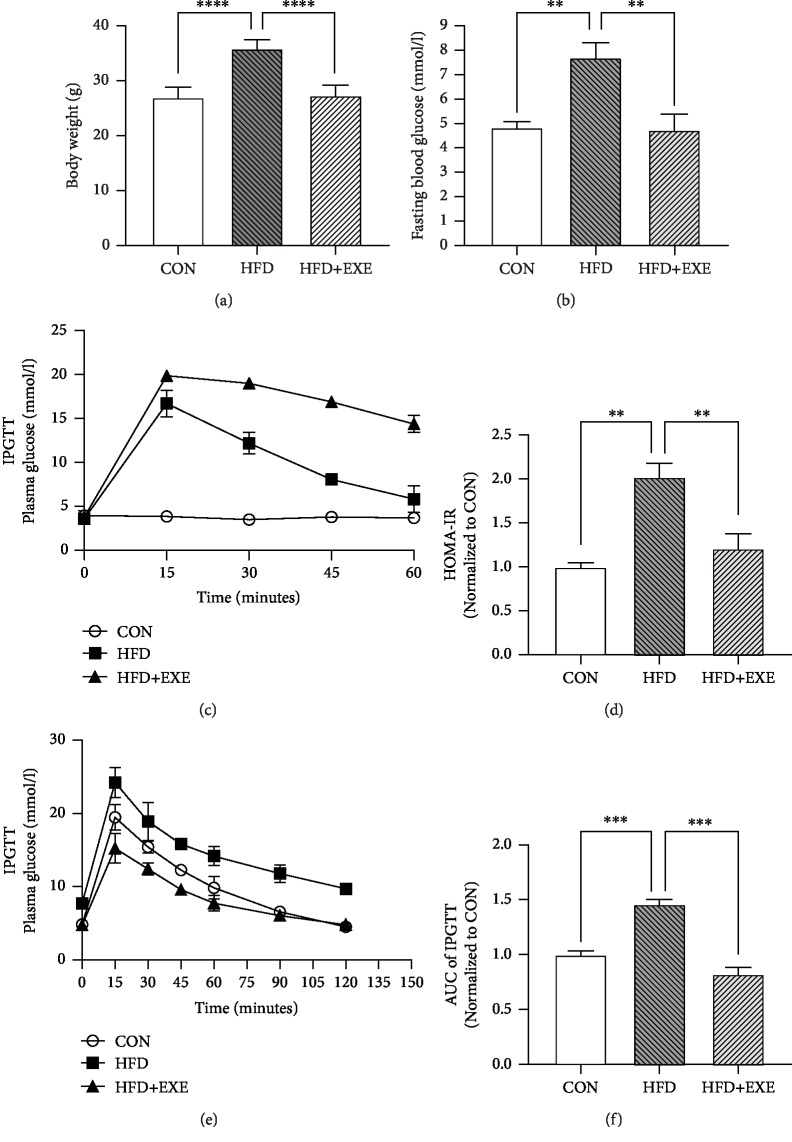
Exendin-4 improves glucose metabolism in T2DM mouse models: (a) body weight; (b) fast blood glucose measurement; (c) plasma insulin measurement after glucose injection; (d) homeostasis model assessment; (e) intraperitoneal glucose tolerance test; (f) area under the curve for IPGTT. Values are the mean ± SEM of 7 animals per group.

A high-fat diet- (HFD-) fed mouse model is robust to mimic metabolic abnormalities in human diabetic patients, including obesity, hyperglycemia, and complications of diabetes [20]. After 24-week dietary intervention, the mean body weights of CON, HFD, and HFD+EXE were measured. Compared to that of CON, the body weight of HFD was increased. Exendin-4 treatment (25 nmol/kg/d) prevented gain of weight by HFD (Figure 1(a)). Blood plasma was collected for glucose metabolic analysis. HFD feeding resulted in a significantly increased level of fasting blood glucose (FBG), while HFD+EXE mice showed identical FBG level with the CON (Figure 1(b)). Exendin-4 treatment also facilitated insulin secretion after glucose injection (Figure 1(c)). The homeostasis model assessment (HOMA-IR) level and IPGTT indicated that Exendin-4 improved glucose tolerance and insulin resistance (Figures 1(d) and 1(e)). The HFD+EXE group showed a reduced area under the curve compared to the other two groups (Figure 1(f)). These results exhibited the metabolic effects for glycemic control of Exendin-4 in a type 2 DM mouse model.

### 3.2. Exendin-4 Protects against Cardiac Dysfunction and Remolding in Diabetic Mice

**Figure 2:**
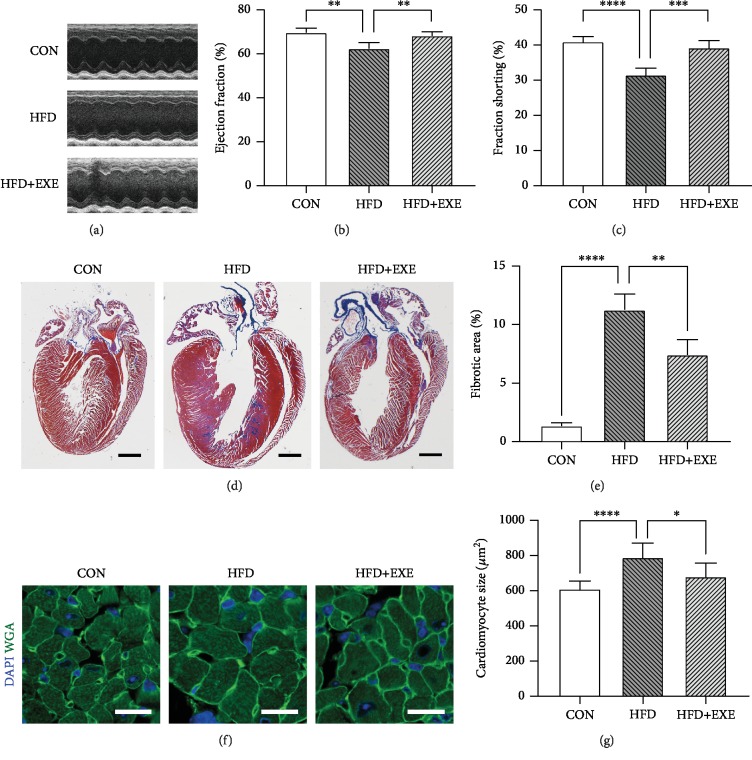
Exendin-4 prevents myocardial remolding and improves cardiac function in diabetic cardiomyopathy: (a) electrocardiogram; (b) ejection fraction; (c) fraction shorting; (d) Masson's trichrome staining of coronal cardiac sections; myocardium blue stain indicating fibrotic tissues (scale bar: 1 mm); (e) quantification of cardiac fibrosis; (f) immunofluorescence of cardiac sections (scale bar: 25 *μ*m; green: WGA and blue: DAPI); (g) transverse area of cardiomyocytes. Values are the mean ± SEM of 5 animals per group.

To determine the cardioprotective role of Exendin-4 in DCM, we studied the cardiac function and histology of the three groups of animals. Echocardiography was performed 7 days prior to sacrifice. Eject fraction (EF) and fractional shorting (FS) were measured for systolic and diastolic function assessment. HFD-fed mice displayed impaired systolic and diastolic function (Figure 2(a)). FS and EF were decreased in HFD compared to CON mice (Figures 2(b) and 2(c)). Exendin-4 treatment significantly rescued HFD-induced cardiac dysfunction (Figures 2(a)–2(c)). Masson's trichrome stain showed thickened left ventricular free walls (Figure 2(d)) and fibrotic deposition in HFD hearts (Figure 2(e)), which implicated pathological cardiac remolding. Treatment of Exendin-4 protected against HFD-induced myocardial hypertrophy and fibrosis (Figures 2(d) and 2(e)). To study the morphology of cardiomyocytes, we stained the heart section with WGA, a widely used dye labeling cell membrane (Figure 2(f)). Quantification of the transverse sectional area of cardiomyocytes indicated that Exendin-4 treatment prevented HFD-driven cardiomyocyte hypertrophy (Figure 2(g)). Taken together, these evidences demonstrated that Exendin-4 protects against cardiac dysfunction and remolding in a type 2 diabetic rodent model.

### 3.3. Exendin-4 Treatment Attenuated Inflammatory Response in the Diabetic Myocardium

**Figure 3:**
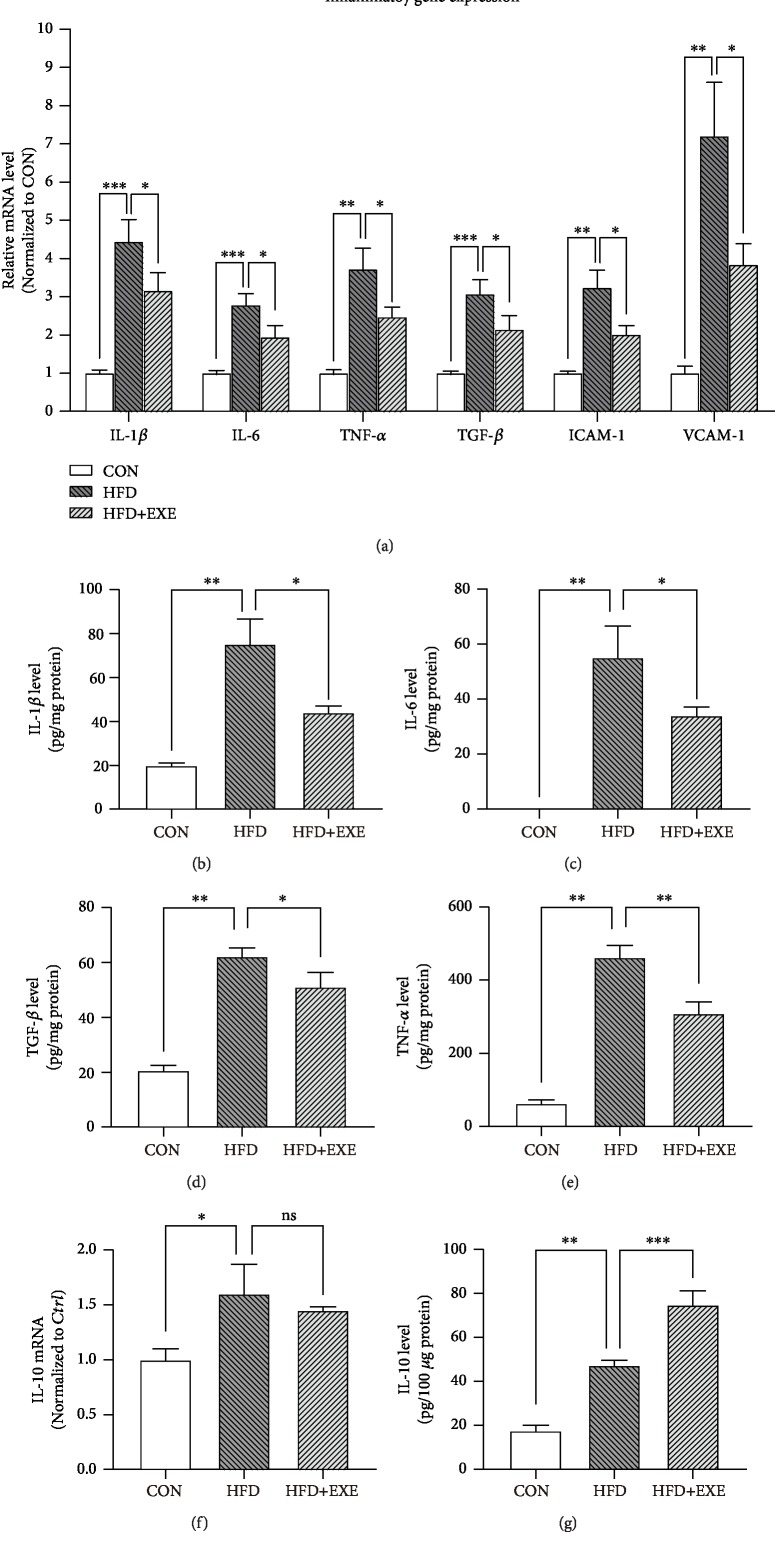
Exendin-4 attenuates inflammatory response in the diabetic myocardium: (a) q-PRC results of inflammatory factors in the myocardium; (b–e) ELISA of proinflammatory cytokines in the heart: IL-1*β* (b), IL-6 (c), TGF-*β* (d), and TNF-*α* (e); (f) q-PRC quantification of *IL-10* in cardiac tissues; (g) IL-10 ELISA. Values are the mean ± SEM of 3 samples per group.

Chronic inflammation involved in cardiac remolding is considered a consequence of cardiomyocyte pyroptosis. To assess the strength of cardiac inflammatory response in the three groups of animals, we performed reverse transcriptase qPCR to quantify the mRNA level of proinflammatory genes, including *IL-1β*, *IL-6*, *TNF-α*, *TGF-β*, *ICAM-1*, and *VCAM-1*. Compared to those in the CON, most proinflammatory genes were upregulated over 3 times in HFD samples. Exendin-4 treatment attenuated the HFD-driven transcriptional activity of these genes (Figure 3(a)). Next, we purified ventricular protein extraction and examined the enrichment of proinflammatory cytokines: *IL-1β*, *IL-6*, *TNF-α*, and *TGF-β*. ELISA results displayed that Exendin-4 negatively regulated the production of these proinflammatory cytokines induced by HFD (Figures 3(b)–3(e)). Next, we examined the level of the anti-inflammatory cytokine IL-10 in heart tissues. qPCR results showed that HFD feeding enhanced the transcriptional activity of *IL-10*, which was not altered by Exendin-4 (Figure 3(f)). However, HFD+EXE samples showed increased protein level of IL-10, compared to the HFD ones (Figure 3(g)). Taken together, these evidences implicated that Exendin-4 attenuated inflammatory response via regulation of both proinflammatory and anti-inflammatory cytokines.

### 3.4. Treatment of Exendin-4 Suppressed the Activation of Caspase-1 in Diabetic Cardiomyocytes

**Figure 4:**
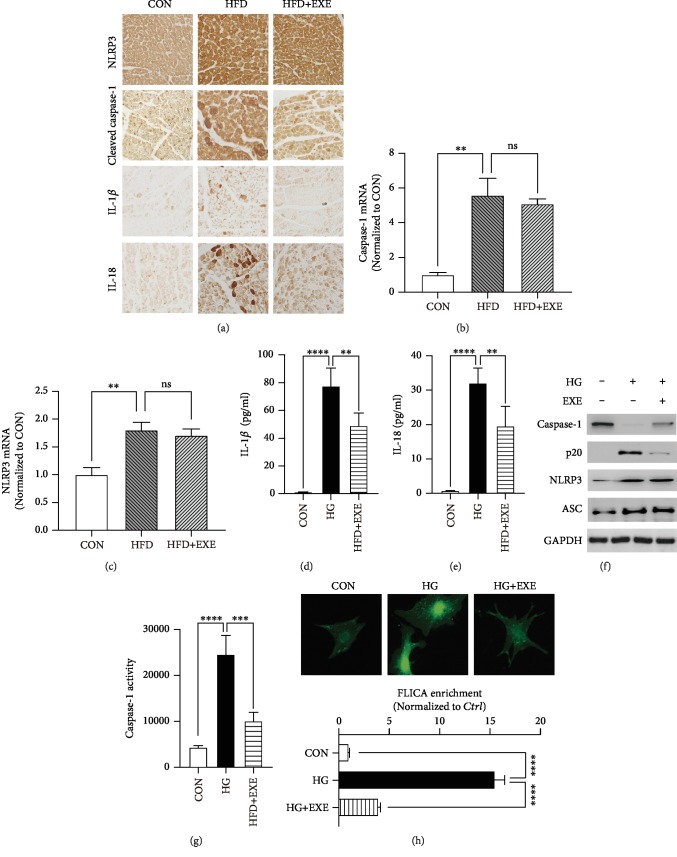
Exendin-4 inhibits diabetic cardiomyocyte pyroptosis: (a) immunohistochemistry of NLRP3, cleaved caspase-1, IL-1*β*, and IL-18 in the myocardium; (b, c) transcriptional activity of *caspase-1* and *NLRP3* in the heart (*n* = 3); (d, e) IL-1*β* and IL-18 ELISA with supernatant of cardiomyocyte culture medium; (f) Western blot of pyroptotic proteins; (g) caspase-1 activity assay (*n* = 3); (h) FLICA staining in cardiomyocytes. Upper panels showed the fluorescent images of CON, HG, and HG+EXE cardiomyocytes. The lower panel is a statistic of the enrichment of FLICA emitting fluorescence in single cells (*n* = 10).

Pyroptosis is a great contributor to cardiac inflammation in DCM [21]. Exendin-4 treatment significantly downregulated cardiac IL-1*β* level induced by HFD feeding. Meanwhile, IL-1*β* is a direct target of caspase-1, the pyroptotic-specific proteinase caspase [11]. Therefore, it is interesting to ask whether Exendin-4 is involved in the regulation of the pyroptotic signaling pathway. We performed immunohistochemistry to visualize the expression level of pyroptotic proteins in the heart, including cleaved caspase-1, NLRP3, IL-1*β*, and IL-18. Compared to the CONs, seriously increased levels of cleaved caspase-1, IL-1*β* and IL-18, and NLRP3 were detected in HFD samples. Exendin-4 treatment suppressed HFD-induced caspase-1 cleavage and decreased the protein levels of IL-1*β* and IL-18, but not NLRP3 (Figure 4(a)). Meanwhile, qPCR results showed no significant difference of *caspase-1* and *NLRP3* mRNA levels between HFD and HFD+EXE samples (Figures 4(b) and 4(c)). These results suggested that Exendin-4 regulated the activation of caspase-1 in the diabetic heart.

To determine the role of Exendin-4 in cardiomyocyte pyroptosis, we examined pyroptotic components in high glucose-treated cardiomyocytes. The primary cardiomyocytes were incubated in the absence (CON) or presence of 40 mM glucose (HG) in combination with Exendin-4 (25 nM) (HG+EXE). Culture medium was collected for quantification of the secretion of IL-1*β* and IL-18 using ELISA. Compared to the CON, HG samples exhibited dramatically increased level of both ILs. Exendin-4 treatment attenuated the increased production of IL-1*β* and IL-18 induced by high glucose (Figures 4(d) and 4(e)). To examine the expression level of caspase-1, we purified protein extraction and performed Western blotting. High glucose facilitated the generation of cleaved caspase-1 (p20) and slightly increased the level of NLRP3 and ASC. Treatment of Exendin-4 inhibited the cleavage of caspase-1 but did not alter the enrichment of NLRP3 and ASC (Figure 4(f)). To determine the activity of caspase-1, firstly, we applied the fluorometric assay based on YVAD-AFC, a substrate of caspase-1 emitting 400 nm light after cleavage. Incubation of cardiomyocytes with high glucose caused significant upregulation of caspase-1 activity, which was diminished by Exendin-4 treatment (Figure 4(g)). The role of Exendin-4 in caspase-1 activation was further identified by the FLICA assay, which labels active caspase-1 in living cells. Quantification of FLICA enrichment in 10 single cells each group confirmed the inhibitory effects of Exendin-4 on caspase-1 activation (Figure 4(h)). In general, these evidences demonstrated that Exendin-4 inhibited the activation of caspase-1 and protected cardiomyocytes against T2DM-induced pyroptosis.

### 3.5. AMPK-TXNIP Pathway Mediates the Antipyroptotic Effects of Exendin-4 in Cardiomyocytes

**Figure 5:**
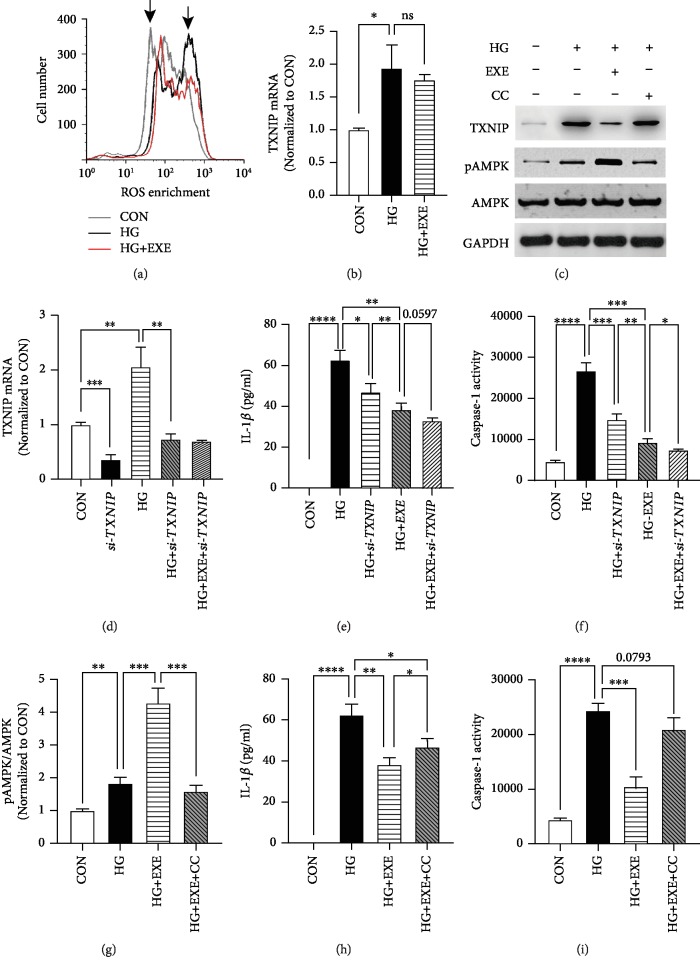
AMPK mediates anti-pyroptotic effects of Exendin-4. (a) ROS determination by FACS. The left arrow indicated the ROS-negative population and the right pointed the positive. (b) Transcription activity of *TXNIP* in cardiomyocytes. (c) Western blot. (d) RNA silencing of TXNIP in cardiomyocytes. (e) IL-1*β* ELISA with TXNIP RNAi. (f) Caspase-1 activity assay with TXNIP RNAi. (g) Quantification of pAMPK/AMPK with CC treatment. (h) IL-1*β* ELISA with CC treatment. (i) Caspase-1 activity assay with CC treatment. Values are the mean ± SEM of 3 samples per group. ^∗^*p* < 0.05, ^∗∗^*p* < 0.01, ^∗∗∗^*p* < 0.005, and ^∗∗∗∗^*p* < 0.001.

In DCM, hyperglycemia-induced ROS generation leads to cardiomyocyte pyroptosis and myocardial inflammation via activating the NLRP3 inflammasome [5, 7]. As an antioxidant, Exendin-4 exhibited cardioprotective effects on multiple cardiac diseases. To determine the antioxidative role of Exendin-4 in high glucose-treated cardiomyocytes, we incubated the cells with fluorescent probe labeling intracellular ROS. Flow cytometry exhibited that high-glucose treatment enlarged the cellular population with high enrichment of ROS. Exendin-4 restored the FACS profile of HG to that of CON (Figure 5(a)). It indicated the antioxidative role of Exendin-4 in high glucose-induced cardiomyocytes.

ROS-driven activation of the NLRP3 inflammasome was characterized by the interaction between NLRP3 and TXNIP [22–25]. As a link between oxidative stress and cardiac inflammation in various cardiovascular diseases [26], TNXIP is a potential regulator involved in the antipyroptotic property of Exendin-4 in high glucose-induced cardiomyocytes. To verify this hypothesis, firstly, we examined the expression level of TXNIP. Incubation of cardiomyocytes in HG medium caused the upregulated mRNA level of *TXNIP*, compared to CON. Exendin-4 treatment did not alter the transcriptional activity of the *TXNIP* gene (Figure 5(b)). However, Western blot showed that Exendin-4 treatment significantly decreased the protein level of TXNIP (Figure 5(c)). It was suggested that TXNIP is posttranscriptionally regulated by Exendin-4 in diabetic cardiomyocytes. Furthermore, we administered cardiomyocytes with *siRNA* targeting TXNIP and examined the pyroptotic activity. RNA silencing of *TXNIP* significantly decreased the expression level of TXNIP (Figure 5(d)) and diminished high glucose-induced caspase-1 activation and IL-1*β* production (Figures 5(e) and 5(f)). It mimicked the inhibitory effects of Exendin-4 on pyroptosis. These results implicated the degeneration of TXNIP as a mediator of Exendin-4 in antipyroptotic regulation.

It is crucial to identify the ROS sensors linking antioxidative and antipyroptotic properties of Exendin-4 in diabetic cardiomyocytes. Previous studies revealed that AMPK is a key regulator of energy metabolism and inflammation in DCM [4, 27, 28]. Activated AMPK degenerates TXNIP to manipulate the activity of the NLRP3 inflammasome [29]. Meanwhile, the activation of AMPK is driven by oxidative stress via ROS-dependent phosphorylation [30]. In high glucose-treated cardiomyocytes, a slight increase in phosphorylated AMPK (pAMPK) was observed. Interestingly, Exendin-4 treatment further facilitated the phosphorylation but did not alter the level of total AMPK (Figures 5(c) and 5(g)). To determine the role of AMPK in antipyroptotic effects of Exendin-4, we pretreated HG+EXE cardiomyocytes with CC (10 *μ*M), a specific AMPK inhibitor, and examined the level of TXNIP and of IL-1*β*. CC treatment increased the protein level of TXNIP (Figure 5(c)) and majorly blocked the inhibitory effect of Exendin-4 on IL-1*β* production (Figure 5(g)). Moreover, the activity of caspase-1 was significantly increased in HG+EXE+CC, compared to HG+EXE (Figure 5(h)). These evidences strongly supported that ROS-sensitive AMPK mediated the antipyroptotic properties of Exendin-4 in hyperglycemia-induced cardiomyocytes.

## 4. Discussion

Exendin-4 has been characterized by cardioprotective effects on cardiovascular diseases, including DCM, in human being [31]. Exendin-4 is involved in apoptosis, autophagy, and various biological processes to promote cardiomyocyte survival [32–34]. As a vital contributor to the development of myocardial inflammation, pyroptosis has not yet been reported to be regulated by Exendin-4 treatment. Our studies revealed the antipyroptotic property of Exendin-4 mediated by the AMPK-TXNIP pathway in hyperglycemia-induced cardiomyocytes.

In a type 2 diabetic mouse model, Exendin-4 treatment mostly rescued cardiac remolding and dysfunction. It has been determined that myocardial inflammation causes loss of cardiomyocytes that eventually results in heart failure [4]. As shown in previous studies [3, 6, 35, 36], diabetic cardiac tissues express increased level of multiple proinflammatory cytokines promoting progression of myocardial inflammation. Exendin-4 treatment negatively regulates the transcription of proinflammatory cytokines and decreased their protein level. In addition, our results showed that the mRNA level of anti-inflammatory IL-10 was increased in the heart of HFD-fed animals. The increased level of IL-10 has also been observed associated with inflammation in various cardiovascular diseases [37, 38]. IL-10 is well characterized as an anti-inflammatory factor and protective factor in myocardial infarction in diabetic animals [39–42]. Therefore, we also proposed that upregulation of IL-10 as a protective mechanism in the HFD-induced diabetic heart attenuates immune response and facilitates cell survival [43]. Exendin-4 treatment did not affect the transcriptional activity of *IL-10* but further increased its protein level. It was suggested that Exendin-4 regulates IL-10 production via a posttranscriptional mechanism. Taken together, we characterized Exendin-4 as anti-inflammatory with clinical potential for DCM treatment.

In the diabetic heart, the NLRP3 inflammasome responds to hyperglycemia-induced toxicity and initiates the progression of pyroptosis [6, 7, 14]. In this study, the protein level of inflammasome components, NLRP3 and ASC, was not affected by Exendin-4-treated hyperglycemia cultured cardiomyocytes, while the upstream activator of inflammasome, TXNIP, was downregulated significantly [22, 24]. The inflammasome is characterized as a molecular dock recruiting procaspase-1 and facilitates its autoactivation [13]. Substantial evidences provided by both *in vivo* and *in vitro* experiments strongly supported that Exendin-4 blocked the activation of caspase-1. Meanwhile, the production of pyroptotic proinflammatory cytokines IL-1*β* and IL-18 was also diminished by Exendin-4. Based on the above evidence, we proposed that Exendin-4 inhibited pyroptosis via blocking the activation of core pyroptotic regulators, inflammasome and caspase-1.

Oxidative stress and the accelerated ROS production induced by high glucose are known to play key roles in the progression of diabetic cardiovascular disease and cardiomyocyte pyroptosis [21]. In this study, Exendin-4 exhibited antioxidative and antipyroptotic effects in high glucose-treated cardiomyocytes. Previous studies revealed that AMPK, as a ROS sensor activated by Exendin-4, protects against cardiac dysfunction in DCM [33, 44]. Our results indicated that activation of AMPK played an important role in the inhibition of caspase-1 activity by Exendin-4 treatment. In our proposal, on the hand, Exendin-4 treatment promotes the activation of AMPK to suppress the activation of the downstream NLRP3 inflammasome and caspase-1. The phosphorylation of AMPK, in response to hyperglycemia-induced oxidative stress, is upregulated as a resistant mechanism against ROS-induced cardiac inflammation. However, in pathological condition, the expression of TXNIP is significantly upregulated that the activity of AMPK is not sufficient for TXNIP degeneration in the absence of Exendin-4. On the other hand, Exendin-4 may facilitate degeneration of TXNIP by improving its sensitivity to AMPK activation. It is known that degeneration of TXNIP is an AMPK-dependent process [29]. Desensitization of TXNIP to AMPK activity may attenuate efficiency of AMPK on TXNIP degeneration. The later hypothesis requires further evidence to prove in future studies.

## 5. Conclusion

Our study revealed that Exendin-4 improved cardiac function and attenuated inflammatory response in the diabetic heart and protected cardiomyocytes against hyperglycemia-induced pyroptosis. The antipyroptotic property of Exendin-4 was mediated by the ROS-AMPK-TXNIP pathway, which regulated the activity of the inflammasome and caspase-1 in diabetic cardiomyocytes.

## Data Availability

All data generated and analyzed during the current study are included in this published article.
